# In silico analysis of promoter region and regulatory elements of glucan endo-1,3-beta-glucosidase encoding genes in *Solanum tuberosum*: cultivar DM 1-3 516 R44

**DOI:** 10.1186/s43141-021-00240-0

**Published:** 2021-09-30

**Authors:** Atnafu Kebede, Mulugeta Kebede

**Affiliations:** 1 Department of Applied Biology, School of Applied Natural Science, Adama Science and Technology University, P.O. Box 1888, Adama, Ethiopia; 2 Department of Biology, College of Natural and Computational Sciences, Dire Dawa University, P.O. Box 1362, Dire Dawa, Ethiopia

**Keywords:** *Solanum tuberosum*, Glucan endo-1,3-beta-glucosidase, CpG island, Motif, Promoter, Transcription factor

## Abstract

**Background:**

Potato (*Solanum tuberosum L*.) is one of the most important food crops in the world. Pathogens remain as one of the major constraints limiting potato productivity. Thus, understanding of gene regulation mechanism of pathogenesis-related genes such as glucan endo-1,3-beta-glucosidase is a foundation for genetic engineering of potato for disease resistance and reduces the use of fungicides. In the present study, 19 genes were selected and attempts were made through in silico methods to identify and characterize the promoter regions, regulatory elements, and CpG islands of glucan endo-1,3-beta-glucosidase gene in *Solanum tuberosum* cultivar DM 1-3 516 R44.

**Results:**

The current analysis revealed that single transcription start sites (TSSs) were present in 12/19 (63.2%) of promoter regions analyzed. The predictive score at a cutoff value of 0.8 for the majority (84.2%) of the promoter regions ranged from 0.90 to 1.00. The locations for 42% of the TSSs were below −500 bp relative to the start codon (ATG). MβGII was identified as the common promoter motif for 94.4% of the genes with an *E* value of 3.5e−001. The CpG analysis showed low CpG density in the promoter regions of most of the genes except for gene ID102593331 and ID: 102595860. The number of SSRs per gene ranged from 2 to 9 with repeat lengths of 2 to 6 bp. Evolutionary distances ranged from 0.685 to 0.770 (mean = 0.73), demonstrating narrower genetic diversity range. Phylogeny was inferred using the UPGMA method, and gene sequences from different species were found to be clustered together.

**Conclusion:**

In silico identified regulatory elements in promoter regions will contribute to our understanding of the regulatory mechanism of glucan endo-1,3-beta-glucosidase genes and provide a promising target for genetic engineering to improve disease resistance in potatoes.

**Supplementary Information:**

The online version contains supplementary material available at 10.1186/s43141-021-00240-0.

## Background

Potato (*Solanum tuberosum L*.) is one of the most widely consumed carbohydrate-rich staple foods in large parts of the world; it is the fourth largest food crop in production [1]. Potato is mainly used as a staple food, but it also has a number of medicinal values. Moderate consumption of the juice from the tubers is used in the treatment of peptic ulcers, bringing relief from pain and acidity [2].

Pathogenesis-related proteins, often called PR proteins, are a structurally diverse group of plant proteins that are toxic to invading fungal pathogens. They are widely distributed in plants in trace amounts, but are produced in much greater concentrations following pathogen attack or stress. PR proteins exist in plant cells intracellularly and also in the intercellular spaces, particularly in the cell walls of different tissues. Varying types of PR proteins have been isolated from each of several crop plants. Different plant organs, e.g., leaves, seeds, and roots, may produce different sets of PR proteins. Different PR proteins appear to be expressed differentially in their hosts in the field when temperatures become stressful, low or high, for extended periods [3].

The several groups of PR proteins have been classified according to their function, serological relationship, amino acid sequence, molecular weight, and certain other properties. PR proteins are either extremely acidic or extremely basic and therefore are highly soluble and reactive. At least 14 families of PR proteins are recognized. Among these pathogenesis-related proteins, glucan endo-1,3-beta-glucosidases (β-1,3-glucanases) are one important hydrolytic enzyme that is abundant in many plant species after infection by different types of pathogens. The amount of them significantly increases and plays a major role in defense reaction against fungal pathogens by degrading the cell wall, because β-1,3-glucan is a structural component of the cell walls of many pathogenic fungi. Glucan endo-1,3-beta-glucosidase appears to be coordinately expressed along with chitinases after fungal infection. This co-induction of the two hydrolytic enzymes has been described in many plant species, including pea, bean, tomato, tobacco, maize, soybean, potato, and wheat [4–11]. In addition to their roles in pathogen defense, glucan endo-1,3-beta-glucosidases have been implicated in cell division, pollen development, pollen tube growth, regulation of plasmodesmata signaling, cold response, seed germination, and maturation [12].

Glucan -1,3-beta-glucosidase forms highly complex and diverse gene families in plants, and a single plant species may have various copies of glucan-1,3-beta-glucosidase genes [12]. The glucan -1,3-beta-glucosidases are the enzymes which can cleave the beta glycosidic linkages of glucans. They can be divided into two groups, exo or endo. The exo-hydrolases catalyze the hydrolysis of the beta-glucan chain by sequentially cleaving glucose residues from the non-reducing end and releasing glucose as the sole hydrolysis product. The endo-hydrolases cleave β-linkages at apparently random sites along the polysaccharide chain, releasing smaller oligosaccharides [13]. The enzyme glucan-1,3-beta-glucosidase is important to delay the growth of pathogenic fungi and to decrease the damage caused by disease in fruits. The application of this enzyme is possible due to the composition of the cell walls of certain microorganisms which contain β-glucans [14].

Many studies have shown that the synthesis of glucan endo-1,3-beta-glucosidase is stimulated when plants are infected by fungal, bacterial, or viral pathogens, and its concentration also increases dramatically. For instance, mRNA for a tomato glucan endo-1,3-beta-glucosidase accumulated to a higher level in leaves infected with the fungal pathogen *Cladosporium fulvum* [15], barley infected with powdery mildew [16], maize infected with *Aspergillus flavus* [17], pepper infected with *Phytophthora capsici*, wheat infected with *Fusarium graminearum* [11], chickpea infected with *Ascochyta rabiei* (Pass.) Labr [18]., and peach infected with *Monilinia fructicola* [19]. Scientists throughout the world have tried to analyze or predict the regulatory elements of pathogen-related genes in higher plants whose expression products have an inhibitory effect on microorganisms such as fungi. However, only a small percentage of PR genes have been investigated.

To the best of our knowledge, there is no report that evaluates the regulatory elements of glucan endo-1,3-beta-glucosidase genes in potato (*Solanum tuberosum* L). Moreover, owing to the crucial roles of glucan endo- 1,3-beta-glucosidase genes in the plant defense system, it is imperative to understand and analyze the promoter region and regulatory elements of glucan endo-1,3-beta-glucosidase genes in *Solanum tuberosum*. The knowledge will contribute to our understanding of the expression profiles and regulatory mechanism of glucan endo-1,3-beta- glucosidase genes. It also provides a promising target for genetic engineering for improved glucan endo-1,3-glucosidase expression in potato and uplifts the level of defense response in potato against fungal pathogens and develops disease-resistant transgenic potato, which is an environmentally friendly approach of a disease control method.

## Methods

A total of 27 whole genome shotgun gene sequences of glucan endo-1,3-beta-glucosidase for *Solanum tuberosum* cultivar DM 1-3 516 R44 were retrieved from the NCBI database available at https://www.nlm.nih.gov/gene; of these, 19 of them were selected for analysis, while the remaining eight gene sequences were excluded from this analysis because they were not having the functional gene structure (many stop codons appear in the middle and the reading frame was highly fragmented), after checking with CLC Genomics Workbench ver. 3.6.1 (http://clcbio.com, CLC bio, Aarhus, Denmark) (Table 1).

**Table 1:** List of the glucan endo-1,3-beta-glucosidase genes of *Solanum tuberosum* cultivar DM1-3 156R44 selected for analysis

S no	GI	Gene name
1	ID: 102588651	Glucan endo-1,3-beta-glucosidase 1-like
2	ID: 102594958	Glucan endo-1,3-beta-glucosidase-like
3	ID: 102601393	Glucan endo-1,3-beta-glucosidase 12-like
4	ID: 102595473	Glucan endo-1,3-beta-glucosidase-acidic isoform G19
5	ID: 102593331	Glucan endo-1,3-beta-glucosidase-like protein 3-like
6	ID: 102578898	Glucan endo-1,3-beta-glucosidase 13 like
7	ID: 102583593	Glucan endo-1,3-beta-glucosidase 11-like
8	ID: 102595860	Glucan endo-1,3-beta-glucosidase 12-like
9	ID:102605560	Glucan endo-1,3-beta-glucosidase, basic isoform 1
10	ID: 102601178	Glucan endo-1,3-beta-glucosidase 4
11	ID:102587248	Glucan endo-1,3-beta-glucosidase 13-like
12	ID: 102604922	Glucan endo-1,3-beta-glucosidase 14 like
13	ID: 102605428	Glucan endo 1,3-beta-glucosidase, acidic isoform PR-Q’-like
14	ID: 102596927	Glucan endo 1,3-beta-glucosidase, acidic isoform PR-Q’
15	ID: 102583800	Glucan endo-1,3-beta-glucosidase 11-like
16	ID: 102581946	Glucan endo-1,3-beta-glucosidase 2-like
17	ID: 102578810	Glucan endo-1,3-beta-glucosidase 12-like
18	ID: 102595638	Glucan endo-1,3-beta-glucosidase-like protein 3-like
19	ID: 102589208	Glucan endo-1,3-beta-glucosidase A

### Finding of transcription start sites and determination of promoter sequence

Glucan endo-1,3-beta-glucosidase gene sequences of *Solanum tuberosum* cultivar DM 1-3 516 R44 were downloaded in FASTA file from NCBI Genome Browser, and 1-kb DNA sequences upstream ATG were used as an input file for determining the transcriptional start sites (TSSs) for the retrieved genes. The Neural Network Promoter Prediction (NNPP version 2.2) tool set was used with the minimum standard predictive score (between 0 and 1) available at https://www.fruitfly.org/seq_tools/promoter.html [20]. For those regions containing more than one TSS, the highest prediction score was considered.

### Motif discovery and comparison of the discovered motif against a database of known motifs

Motif discovery was performed by MEME suite (Multiple Em for Motif Elicitation) software version 3.5.4 available at http://meme-suite. org/tools/meme using minimum and maximum motif width of 6 and 50 bp, respectively, and a maximum number of 3 motifs; the rest of the parameters were kept at default. The MEME output was shown in HTML, as well as in several other formats. The motif with the least *E*-value was used for comparison against a database of known motifs using TOMTOM and ranked the motifs in the database and produce an alignment for each significant match [21]. TOMTOM reported for each query a list of target motifs, ranked by *p*-value and *q*-value of each match [22]. TOMTOM also displayed putative transcription factors (TFs) that resemble the TFs of glucan endo-1,3-beta-glucosidase genes. Finally, after identification of those putative TFs interacting with DNA motif, the role of the TFs was described.

### CpG island analysis

Sequences of 2000 bp upstream ATG for each glucan endo-1,3-beta-glucosidase gene of *Solanum tuberosum* cultivar DM 1-3 516 R44 were downloaded in FASTA format from NCBI (https://www.ncbi.nlm.nih.gov/), and the bioinformatics prediction of CpG islands was analyzed using CLC Genomics Workbench ver. 3.6.1 (available at http://clcbio.com, CLC bio, Aarhus, Denmark). Searching for *MspI* cutting sites (fragment sizes between 40 and 220 bp) is relevant for the detection of CGIs, because studies using whole genome CpG island libraries prepared for different species revealed that CpG islands are not randomly distributed but are concentrated in particular regions, because CpG-rich regions are achieved by isolation of short fragments after *MspI* digestion that recognizes CCGG sites [23]. The parameter setting was as follows, with a guanine and cytosine (GC) content greater than or equal to 55% and observed to expected CpG ratio (Obs CpG/ExpCpG) greater than or equal to 0.65 and length ≥500 bp [24].

### Mining glucan endo-1,3-beta-glucosidase genes for simple sequence repeats

The 19 query sequences of glucan endo-1,3-beta-glucosidase genes of *Solanum tuberosum* cultivar DM 1-3 516 R44 were screened to detect di-, tri-, tetra-, penta-, and hexanucleotide simple sequence repeat (SSR) motifs using the SSRIT tool available at Gramene database (http://www.gramene.org/db/searches/ssrtool). After a thorough examination, the output was generated with details of the repeat motif, number of repeat units, repeat length, SSR start, and SSR end point [25].

### Phylogenetic relationship analysis

The phylogenetic analysis was inferred using the UPGMA method [26]. The analysis involved 40 glucan endo-1,3-beta-glucosidase gene sequences selected from *Solanum tuberosum*, *Nicotiana tabacum*, *Solanum lycopersicum*, and *Arabidopsis thaliana* [26]. The genetic distances were computed using the p-distance method [27]. Codon positions included were 1st+2nd+3rd+Noncoding. All ambiguous positions were removed for each sequence pair (pairwise deletion option). The phylogenetic analysis, genetic distances, conserved sites, variable sites, and base composition of the gene sequences were conducted using the Molecular Evolution Genetic Analysis X32 (MEGA X32) available at https://www.megasoftware.net/ [28].

## Results

### Finding of transcription start sites and determination of promoter sequence

Transcription start sites (TSSs) predicted for each of the 19 study subjects are presented in Table 2. The prediction showed that the glucan endo-1,3-beta-glucosidase genes of *Solanum tuberosum* cultivar DM 1-3 516 R44 had TSSs ranging from 1 to 3. The predictive score for the majority 16 (84.2%) of the promoter regions was 0.90 and above. The highest promoter prediction score (1.0) was obtained for two gene sequences only (Pro-102604922 and Pro-102581946) while the lowest promoter prediction score (0.8) was obtained in none of them (Table 2). In addition, the result of promoter predictions for glucan endo-1,3-beta-glucosidase gene sequences with a cutoff value of 0.80 showed that the majority 12 (63.2%) of the gene sequences showed only one TSS, while 7 (36.8%) of them revealed multiple TSSs.

**Table 2:** Number and predictive score for glucan endo-1,3-beta-glucosidase genes of *Solanum tuberosum* cultivar DM 1-3 156 R44 TSSs

Gene ID	Corresponding promoter region name	Number of TSS identified	Predictive score at a cutoff value of 0.8	Location of the best TSS upstream of the translation start site
ID102588651	Pro-102588651	1	0.99	−849
ID102594958	Pro-102594958	3	0.81, 0.84, **0.98**	−277
ID102601393	Pro-102601393	1	0.94	−79
ID102595473	Pro-102595473	1	0.91	−724
ID102593331	Pro-102593331	1	0.98	−379
ID102578898	Pro-102578898	1	0.98	−2900
ID102583593	Pro-102583593	3	0.82, 0.84, **0.91**	−79
ID102595860	Pro-102595860	1	0.94	−1579
ID102605560	Pro-102605560	2	0.81, **0.93**	−522
ID102601178	Pro-102601178	1	0.90	−2125
ID102587248	Pro-102587248	1	0.91	−50
ID102604922	Pro-102604922	3	0.82, 0.93.**1.00**	−1402
ID102605428	Pro-102605428	1	0.88	−313
ID102596927	Pro-102596927	2	0.82, **0.99**	−429
ID102583800	Pro-102583800	1	0.81	−348
ID102581946	Pro-102581946	1	1.00	−694
ID102578810	Pro-102578810	3	0.86, 0.94, **0.97**	−1880
ID102595638	Pro-102595638	3	0.83, 0.85, **0.93**	−751
ID102589208	Pro-102589208	1	0.87	−686

^a^NNPP tool prediction result is considered reliable at 0.8 cutoff values for eukaryote organism [20]. Values in bold are the highest prediction scores for sequences having multiple TSS

In general, the TSSs of gene sequences were located between the range of −79 and −2900 bp relative to the translation start codon (ATG), with a relatively highest occurrence in the region above −1000 bp (5 sequences), followed by −201 to −400 bp and -601 to −800 bp regions (4 sequences, each), −1 to −200 bp (3 sequences), and −401 to −600 (2 sequences), while the lowest occurrence was observed at −801 to −1000 bp (1 sequence).

### Discovery of common motifs and associated TFs in the promoter regions 

**Fig. 1:**
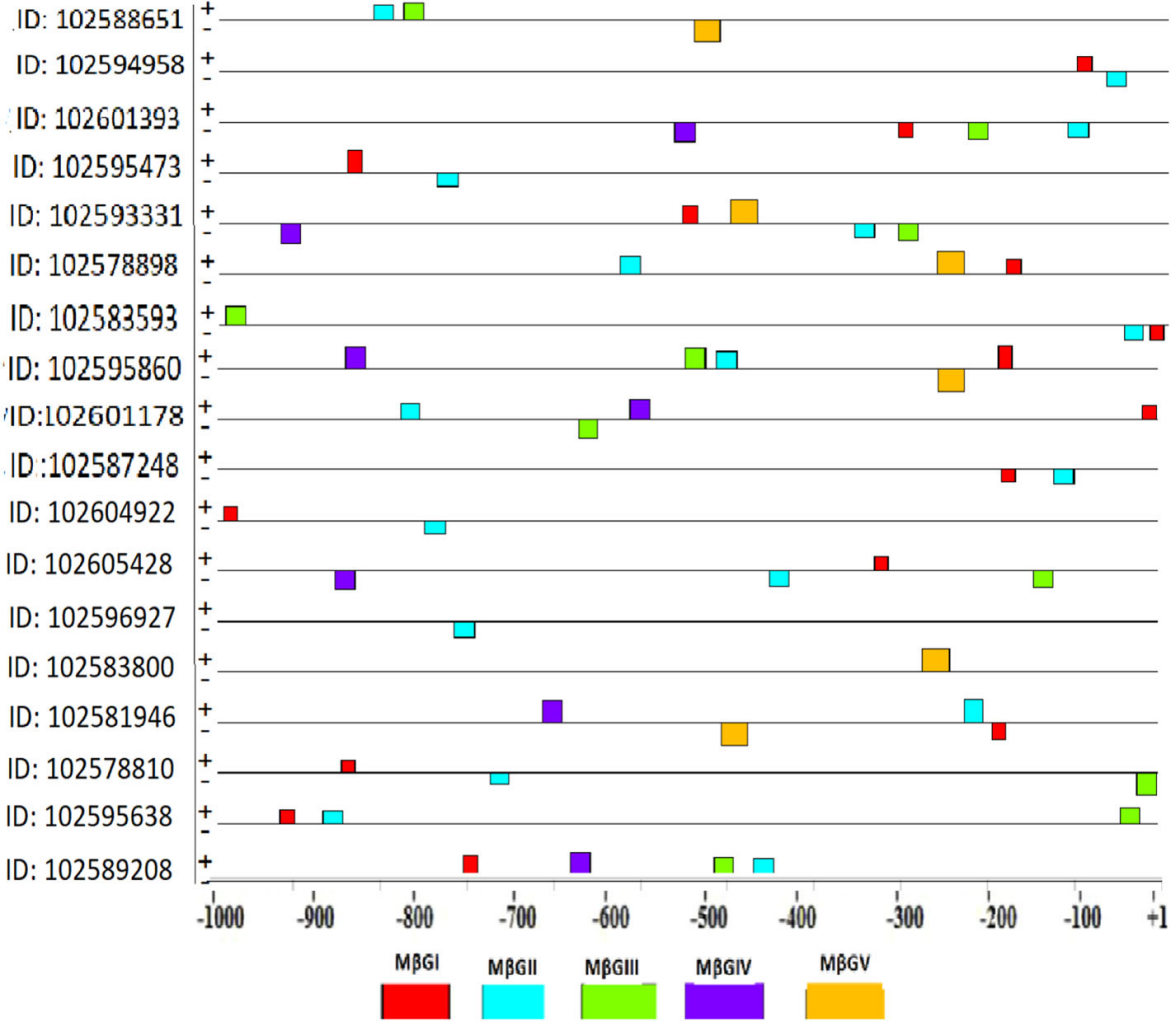
The discovered motifs in glucan endo-1,3-beta-glucosidase genes of *Solanum tuberosum* cultivar DM 1-3 516 R44

In the current study, five candidate motifs that were shared by glucan endo-1,3-beta-glucosidase gene promoter sequences of *Solanum tuberosum* cultivar DM 1-3 516 R44 were discovered (Table 3). The relative location and spatial distribution of the majority of the discovered common motifs were concentrated between +1 and −500 bp of the TSSs. MEME generated common candidate motifs for 18/19 of the gene promoter sequences. It is also interesting to notice that the discovered motifs were distributed on both positive and negative strands with 30 and 25, respectively, as shown in Fig. 1.

**Table 3:** Identified common candidate motifs in *Solanum tuberosum* DM 1-3 156 R44 glucan endo-1,3- beta-glucosidase gene promoter regions

Discovered candidate motif	Number (%) of beta 1,3-glucosidase promoters containing each one of the motifs	***E***-value^**a**^	Motif width	Total no. of binding sites
MβGI	15 (83.3%)	3.6e−010	15	15
MβGII	17 (94.4%)	3.5e−001	21	17
MβGIII	10 (55.5%)	4.9e+000	21	10
MβGIV	7 (38.8%)	9.6e+002	21	7
MβGV	6 (33.3%)	7.7e+002	28	6

^a^Probability of finding an equally well-conserved motif in random sequences

**Fig. 2:**
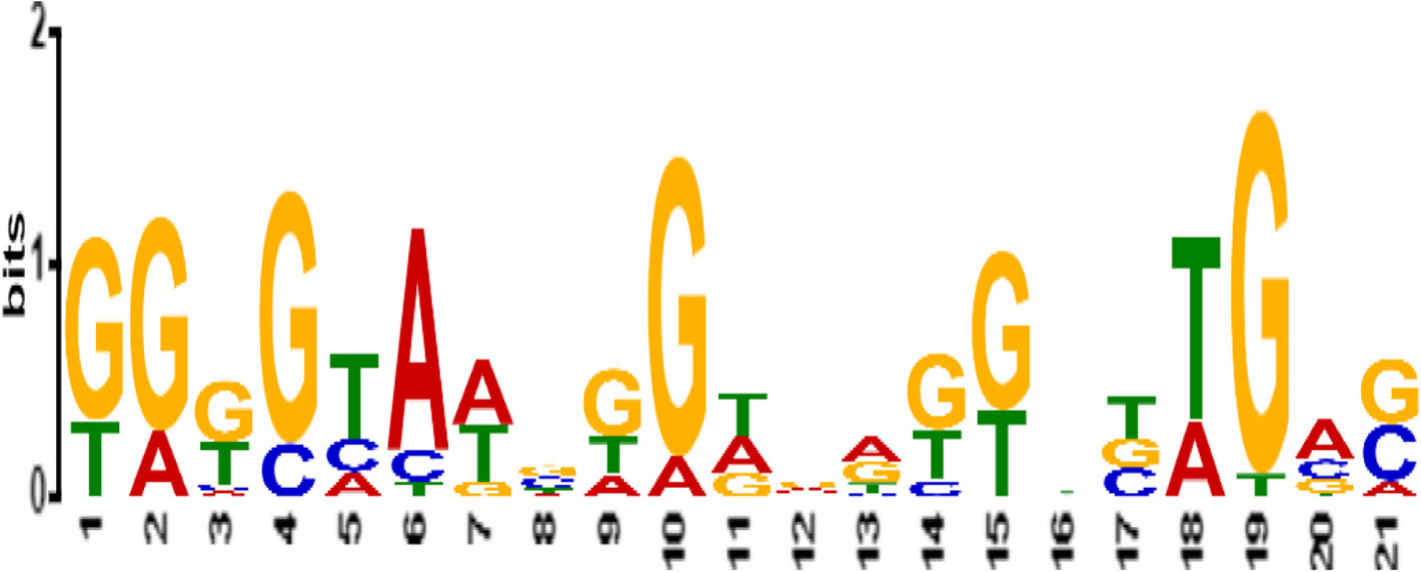
Sequence logo for the identified common motif MβGII for glucan endo-1,3-beta-glucosidase genes of *Solanum tuberosum* cultivar DM1-3 156 R44

To determine a candidate common promoter motif which is functionally important, a motif which was shared by the majority of promoter regions of *Solanum tuberosum* glucan endo-1,3-beta-glucosidase genes was selected. Among the five motifs, MβG II was identified as a common promoter motif shared by 94.4% of *Solanum tuberosum* glucan endo-1,3-beta-glucosidase promoters. A common promoter motif serves as binding sites for transcription factors involved in gene expression and regulation of these genes. A sequence logo for MβGII generated by MEME is presented in Fig. 2. Moreover, further analysis was carried out to get more information on the MβGII motif of the potato (*Solanum tuberosum* DM 1-3 156 R44) glucan endo-1,3-beta-glucosidase genes. Thus, MβGII was compared to registered motifs in publicly available databases to see if they are similar to known regulatory motifs.

### Discovery of matches to the query motif

Among the discovered five common candidate motifs, MβGII with the *E* value of 3.5e−001 was used as a query motif for comparison against a database of JASPAR2018_CORE_vertebrates non-redundant uniprobe_mouse of known motifs using TOMTOM web application [21]. The analysis showed that the query motif MβGII serves as binding sites for 8 transcription factors, namely, (MA0016.1(usp), MA0359.1(RAP1), MA0159,1(RARA: RXRA), MA1149.1 (RARA: RXRG), MA0258.2(ESR2), UP00070_2(Gcm1_ secondary), MA0450.1(hkb), and MA0801.1(MGA). As we tried to check the role of the identified TFs in the UniProt protein database, they act as a receptor to their target ligands, regulate gene expression in various biological processes and developments, involved in cell adhesion and cell junction formation, and act as a repressor or activator (Table 4).

**Table 4:** List of matches to the query motif from the database JASPAR2018_CORE_vertebrates_non redundant and Uniprobe mouse

S no	Match name	Data base	***E***-value	Over lap	Offset	Orientation	Function
1	(MA0016.1(usp)	JASPAR2018_CORE_vetebrates_non redundant	1.91e−01	10	0	Normal	Receptor for ecdysone. May be an important modulator of insect metamorphosis. Plays an important part in embryonic and post-embryonic development
2	MA0359.1(RAP1)	JASPAR2018_CORE_vetebrates_non redundant	7.76e−01	10	−2	Reverse complement	Rap1 is predominantly involved in cell adhesion and cell junction formation.
3	MA0159,1(RARA:: RXRA)	JASPAR2018_CORE_vetebrates_non redundant	1.25e+00	17	−1	Normal	Receptor for retinoic acid. Retinoic acid receptors bind as heterodimers to their target response elements in response to their ligands, all-trans or 9-cis retinoic acid, and regulate gene expression in various biological processes.
4	MA1149.1 (RARA :: RXRG)	JASPAR2018_CORE_vetebrates_non redundant	2.24e+00	18	0	Normal	Receptor for retinoic acid. Retinoic acid receptors bind as heterodimers to their target response elements in response to their ligands, all-trans or 9-cis retinoic acid, and regulate gene expression in various biological processes
5	MA0258.2 (ESR2)	JASPAR2018_CORE_vetebrates_non redundant	3.76e+00	15	−1	Reverse complement	Its molecular function is transcription, transcription regulation
6	UP00070_2(Gcm1_secondary)	Uniprobe mouse	6.48e+00	17	0	Normal	The transcription factor glial cells missing 1 (*Gcm1*) plays a pivotal role in labyrinth development
7	MA0450.1(hkb)	JASPAR2018_CORE_vetebrates_non redundant	9.09e+00	9	−11	Normal	As a repressor, hkb assures that the formation of mesoderm (by ventral invagination of the presumptive mesoderm) does not spread to the two poles of the egg.
8	MA0801.1 (MGA)	JASPAR2018_CORE_vetebrates_non redundant	9.30e+00	8	−12	Normal	Functions as a dual-specificity transcription factor, regulating the expression of both MAX-network and T-box family target genes. Functions as a repressor or an activator.

### CpG island analysis

In the present study, CpG island analysis of the promoter region was investigated using in silico digestion method (using restriction enzyme *MspI*) and the result showed low CpG density in the investigated regions. Fragments were observed only in gene ID: 102593331 and 102595860 (Table 5). The presence of low-density CpG islands might be associated with selective gene expression at a specific tissue.

**Table 5:** *MspI* cutting sites and fragment sizes for glucan endo -1,3-beta-glucosidase genes in the promoter regions

Region	Gene ID of the corresponding glucan-1,3-beta-glucosidase gene	Nucleotide positions of ***MspI*** sites	Fragment sizes (between 40 and 220 bps)
Promoter region	ID: 102588651	No restriction	–
	ID: 102594958	No restriction	–
	ID: 102601393	No restriction	–
	ID: 102595473	No restriction	–
	ID: 102593331	Restrictions found (at 155 and 1440)	155
	ID: 102578898	No restriction	–
	ID: 1025835931	Single restriction (at 919)	–
	ID: 102595860	Restrictions found (at 1062, 1066, 1134, 1153, and 1318)	68, 165
	ID:102605560	No restriction	–
	ID: 102601178	Single restriction (at 411)	–
	ID:102587248	No restriction	–
	ID: 102604922	No restriction	–
	ID: 102605428	Single restriction (at 1000)	–
	ID: 102596927	No restriction	–
	ID: 102583800	No restriction	–
	ID: 102581946	Single restriction (at 850)	–
	ID: 102578810	No restriction	–
	ID: 102595638	No restriction	-
	ID: 102589208	Single restriction (at 815)	–

### SSR motif occurrence in sequences

In the present study, 265 different SSR motifs ranging in size from 2 to 6 (dimer to hexamer) and in number from 2 to 9 per gene were detected in the gene sequences of *Solanum tuberosum* cultivar DM 1-3 516 R44 examined, shown in supplementary table 1. Dimer motifs such as ac, at, ag, ca, ct, ga, gt, ta, and tc were found in the majority (95%) of the gene sequences. Assuming the presence of a large number of tandem repeats, their effects are likely to occur in the glucan endo-1,3-beta-glucosidase gene of *Solanum tuberosum* cultivar DM 1-3 516 R44. Gene sequences with the highest number of dimer repeats are shown in Table 6.

**Table 6:** Gene sequences with the highest number of dimer repeats

Sequence	Motif	No. of repeats	SSR start	SSR end	Seq length
ID: 102578898	ac	7	4361	4374	4566
ID: 102595860	ta	9	1419	1436	2570

### Genetic divergence among gene sequences from different plant species

The genetic distance was assessed using 40 gene sequences (supplementary table 2). A total of 5812 positions or sites were found in the final dataset. The genetic distance among the gene sequences ranged from 0.685 to 0.770. Gene ID:102605428 and ID:102578810 recorded the least genetic distance (0.685); both are from the same species *Solanum tuberosum*. Meanwhile, the highest genetic distance (0.77) was estimated between ID:102581946 in *Solanum tuberosum* and ID:832156 in *Arabidopsis thaliana* and between ID:107820469 in *Nicotiana_tabacum* and ID:834215 in *Arabidopsis thaliana*, each. The overall mean genetic distance was calculated as 0.73, and this shows a narrower genetic diversity range among the sequences. The distance matrix is shown in supplementary table 3.

### Phylogenetic relationships of glucan endo-1,3-beta-glucosidase gene sequences

**Fig. 3:**
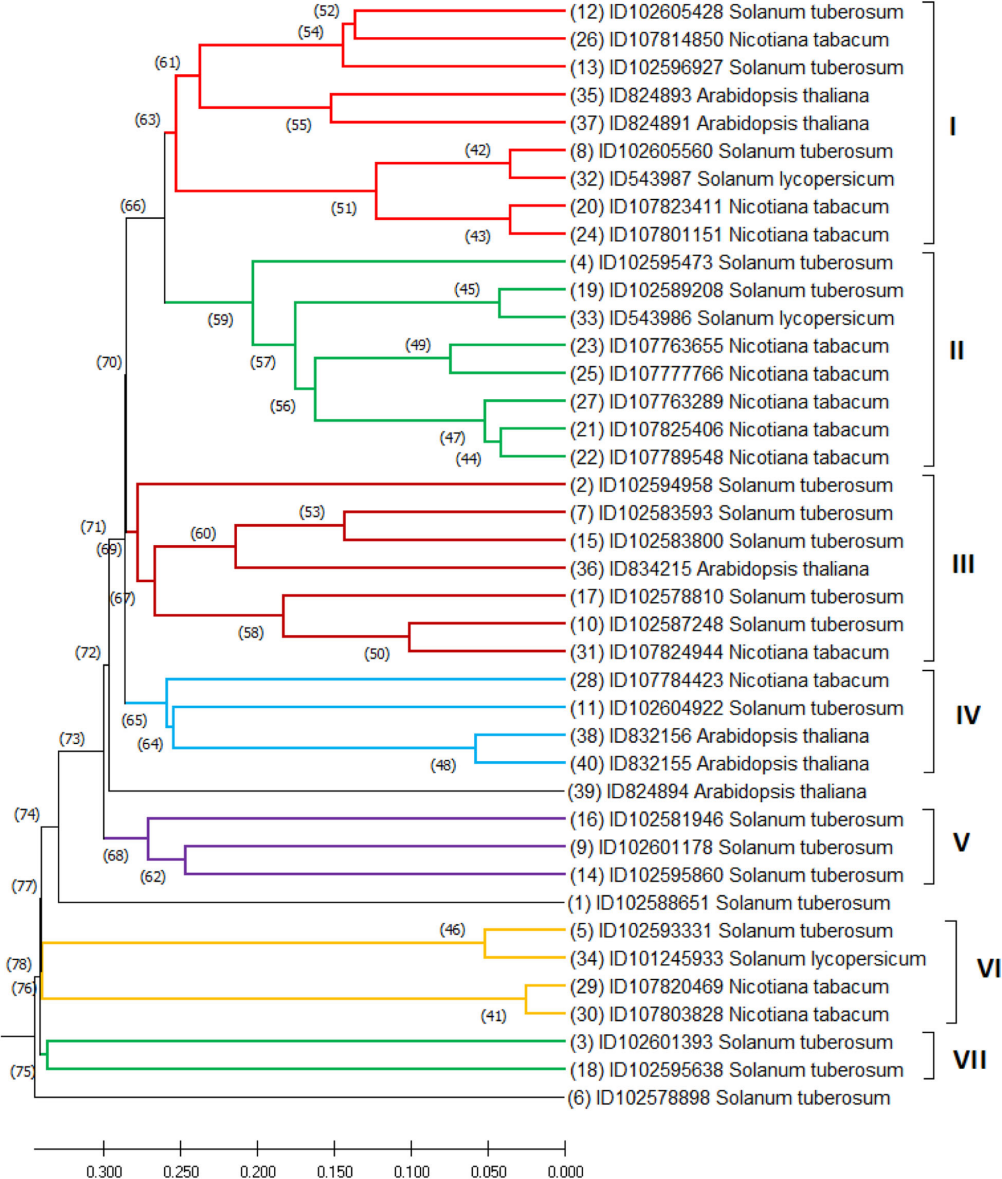
UPGMA phenogram illustrating the relationships among the glucan endo-1,3-beta-glucosidase gene sequences grouped by gene ID and scientific name

The phylogenetic tree resulted in seven clusters: cluster I comprised of 9 gene sequences, 3 from *Nicotiana tabacum*, 2 from *Arabidopsis thaliana*, 3 from *Solanum tuberosum*, and 1 from *Solanum lycopersicum*; cluster II comprised of 8 gene sequences, 5 from *Nicotiana tabacum*, 2 from *Solanum tuberosum*, and 1 from *Solanum lycopersicum*; cluster III comprised of 7 gene sequences, 5 from *Solanum tuberosum*, 1 from *Nicotiana tabacum*, and another 1 from *Arabidopsis thaliana*; cluster IV comprised of 4 gene sequences, 2 from *Arabidopsis thaliana*, 1 from *Nicotiana tabacum*, and 1 from *Solanum tuberosum*; cluster V consisted of 3 gene sequences entirely from *Solanum tuberosum*; cluster VI comprised of 4 gene sequences, 2 from *Nicotiana tabacum*, 1 from *Solanum lycopersicum*, and 1 from *Solanum tuberosum*; and cluster VII comprised of 2 gene sequences mainly from *Solanum tuberosum.* Meanwhile, two gene sequences from *Solanum tuberosum* and one from *Arabidopsis thaliana* were individually isolated from the clusters (Fig. 3).

### Multiple sequence alignment of the gene sequences

The multiple sequence alignment was conducted using the Clustal Omega algorithm available online at https://www.ebi.ac.uk/Tools/msa/. The result ranges from 24.4% (between ID107820469 and ID102605428) to 95.2% (between ID107803828 and ID107824944) shown in supplementary table 4. The number of conserved sites, variable sites, and the frequency of nucleotide bases is mentioned in Table 7. Gene ID102601178 in *Solanum tuberosum* had the lowest rate for both conserved sites and variable sites, accounting for 7.5% and 20.7%, respectively, whereas gene ID102589208 in *Solanum tuberosum* had the greatest value (28.8%) for conserved sites and gene ID832156 in *Arabidopsis thaliana* had the highest proportion (76.1%) for variable sites.

**Table 7:** Number of conserved sites, variable sites, and frequency of each nucleotide

Gene	bp	Conserved site	Variable site	T	C	A	G
ID102588651 *Solanum tuberosum*	1645	410 (24.9%)	1235 (75%)	34.7	17.8	29	18.3
ID102594958 *Solanum tuberosum*	2928	415 (14.1%)	1230 (42%)	33.7	17.4	30	18.7
ID102601393 *Solanum tuberosum*	1969	461 (23.4%)	1184 (60%)	34.5	20.1	29.7	15.5
ID102595473 *Solanum tuberosum*	1587	418 (26.3%)	1169 (73%)	34.4	15.8	32.7	17
ID102593331 *Solanum tuberosum*	3721	447 (12%)	1198 (32.1%)	36	16.6	30.7	16.5
ID102578898 *Solanum tuberosum*	4566	481 (10.5%)	1164 (25.4%)	35.7	17.7	27.8	18.6
ID102583593 *Solanum tuberosum*	1378	369 (26.7%)	1009 (73.2%)	29.3	26.6	25.1	18.8
ID102605560 *Solanum tuberosum*	1545	438 (28.3%)	1107 (71.6%)	32.1	17.8	30.2	19.7
ID102601178 *Solanum tuberosum*	5812	441 (7.5%)	1204 (20.7%)	36.7	16.8	26.9	19.4
ID102587248 *Solanum tuberosum*	1740	388 (22.2%)	1257 (72.2%)	31.6	20	26.8	21.4
ID102604922 *Solanum tuberosum*	5363	432 (8%)	1213 (22.6%)	37	17.5	25.8	19.5
ID102605428 *Solanum tuberosum*	1360	374 (27.5%)	986 (72.5%)	29.2	20.6	31.1	18.8
ID102596927 *Solanum tuberosum*	2460	444 (18%)	1201 (48.8%)	32.6	18.1	31.6	17.6
ID102595860 *Solanum tuberosum*	2570	421 (16.3%)	1224 (47.6%)	33.8	16.7	30.9	18.4
ID102583800 *Solanum tuberosum*	1920	446 (23.2%)	1199 (62.4%)	31.9	22.5	25.2	20.2
ID102581946 *Solanum tuberosum*	3960	434 (10.9%)	1211 (30.5%)	34.2	18.6	27.8	19.2
ID102578810 *Solanum tuberosum*	2778	440 (15.8%)	1205 (43.3%)	35.4	19.3	25.8	19.4
ID102595638 *Solanum tuberosum*	3982	431 (10.8%)	1214 (30.4%)	38.8	16.8	27.8	16.3
ID102589208 *Solanum tuberosum*	1608	464 (28.8%)	1144 (71.1%)	34.5	16.7	32.7	15.9
ID107823411 *Nicotiana tabacum*	2207	456 (20.6%)	1189 (53.8%)	33.6	18.5	29.7	18
ID107825406 *Nicotiana tabacum*	1967	465 (23.6%)	1180 (59.9%)	34.1	18	29.8	17.9
ID107789548 *Nicotiana tabacum*	1814	435 (23.9%)	1210 (66.7%)	35	17.9	30.3	16.7
ID107763655 *Nicotiana tabacum*	2012	410 (20.3%)	1235 (61.3%)	34.2	18.2	31.2	16.2
ID107801151 *Nicotiana tabacum*	2189	461 (21%)	1184 (54%)	34	18.5	29.5	17.8
ID107777766 *Nicotiana tabacum*	2034	445 (21.8%)	1200 (58.9%)	34.1	17.7	31.9	16.1
ID107814850 *Nicotiana tabacum*	1809	466 (25.7%)	1179 (65.1%)	29.1	19.5	30.4	20.7
ID107763289 *Nicotiana tabacum*	1671	437 (26.1%)	1208 (72.2%)	34.3	18.6	30.5	16.4
ID107784423 *Nicotiana tabacum*	1630	432 (26.5%)	1198 (73.4%)	34.4	19.1	28.2	18.2
ID107820469 *Nicotiana tabacum*	1311	342 (26%)	969 (73.9%)	37.2	15	29.9	17.8
ID107803828 *Nicotiana tabacum*	2607	411 (15.7%)	1234 (47.3%)	33.1	19.6	27.7	19.4
ID107824944 *Nicotiana tabacum*	1305	332 (25.4%)	973 (74.5%)	28.4	24.5	26.2	20.7
ID543987 *Solanum lycopersicum*	2025	453 (22.3%)	1192 (58.8%)	34.5	16.7	31	17.6
ID543986 *Solanum lycopersicum*	1717	479 (27.8%)	1166 (67.9%)	34.6	16.5	33.1	15.6
ID101245933 *Solanum lycopersicum*	3858	452 (11.7%)	1193 (30.9%)	37.5	16.5	30.5	15.2
ID824893 *Arabidopsis thaliana*	1571	423 (26.9%)	1148 (73%)	27.6	20.9	29.4	22
ID834215 *Arabidopsis thaliana*	2506	423 (16.8%)	1222 (48.7%)	30.4	24.8	24.7	19.9
ID824891 *Arabidopsis thaliana*	1503	430 (28.6%)	1073 (71.3%)	28	22.5	28.1	21.2
ID832156 *Arabidopsis thaliana*	1140	272 (23.8%)	868 (76.1%)	26.8	24.2	28	20.7
ID824894 *Arabidopsis thaliana*	1953	459 (23.5%)	1186 (60.7%)	31.6	18.5	32.4	17.3
ID832155 *Arabidopsis thaliana*	1602	409 (25.5%)	1193 (74.4%)	28.3	22.6	28.9	20

## Discussion

Finding of transcriptional start site (TSS) triggers the prediction of the promoter region and thus simplifies the subsequent analysis of gene expression. In the present in silico analysis, the number of TSSs per gene sequences was 1 to 3, and the majority 12 (63.1%) of the gene sequences had a single transcription start site, consistent with the previous finding by [29], who reported that 62.1% of the gene sequences contained single TSS. However, in most in silico analysis studies, it has been reported that most genes have more than one TSS [30–34]. In the present study, it was also revealed that the locations for 42% of the TSSs were below −500 bp relative to the ATG. However, several authors reported that the location of the TSSs of the majority (>50%) of the gene sequences studied was below −500 bp relative to ATG [35–38].

Patterns of gene expression (conditionally or temporally) have been linked to transcription regulation [39]. The common promoter motif is short DNA segments that serve as binding sites for TFs involved in gene expression regulation [31]. In the present study, the common promoter motif was found in 18 (94.4%) of the promoter sequences investigated. Some studies reported the sharing of a common promoter motif by all the promoter sequences (100%) [29, 32]. The discovery of matches to the query sequence showed that the query motif serves as binding sites for 8 transcription factors, involved in the regulation of gene expression as a receptor, transcription factor, or repressor in various biological processes (Table 4).

Several studies reported that CpG islands (CGIs) play an important role in the regulation of gene expression [40]. DNA of plant species has been shown to contain more CpG dinucleotides than human DNA [41]. Methylation of cytosine at CpG islands has been shown to restrict the access of promoter region of genes to their transcription factors, hence preventing their expression [42]. Consistent with the present analysis, low CpG content was reported in the promoter region of rice PR2 (beta 1,3-glucanase) genes but none is identified in the promoter region of all the families of *Arabidopsis thaliana* PR gene families [43]. The absence of CpG islands in glucan endo-1,3-beta-glucosidase gene (PR2) might be indicative of tissue-specific gene expression. Ferguson and Jiang [44] also showed that dicots such as potato genome contain low CpG density than monocots. Conversely, Gardiner-Garden and Frommer [45] reported that, in plants, high-density CpG islands tended to lie near the 5′-ends (towards the promoter region) of housekeeping genes which is associated with broad expression of these genes.

In the current study, the cluster analysis showed that the gene sequences from different plant species clustered together. In our results, the range of conserved sites was between 7.5 and 28.8% while the range of variable sites was between 20.7 and 76.1%. Though the percentage range of variable sites was wider than the conserved sites, the phylogeny showed the opposite relationship.

In the present study, the SSR motifs ranged in size from 2 to 6 (dimer to hexamer), and the number of SSR motifs per gene ranged from 2 to 9. The SSR motif analysis also revealed that there is lack of significant variation in the repetition number of the SSR motifs between gene sequences of the different plant species and lack of differences within the repetitive SSR motifs between gene sequences within species. As it is already known, the presence of SSRs within genes can lead to (i) a gain or loss of gene function, (ii) affect transcription and translation, (iii) mRNA splicing, or (iv) export to the cytoplasm. All these effects eventually lead to phenotypic changes [42]. Most often, the length of the simple sequence repeat (SSR) motif does not exceed nine nucleotides and is referred to as short tandem repeats (STRs) or SSRs, or microsatellites. Short tandem repeats are associated with a higher frequency of mutation, affecting DNA sequence composition and length [46].

CGIs are known to concentrate near the transcription start sites (TSSs) of genes. Genes that possess CGIs are often highly expressed in multiple tissues. In the current study, CpG island analysis of the promoter region showed a low density of CpG islands. Possibly, low CpG island density could be one reason for the lack of divergence between gene sequences. According to Prendergast et al. [47], CpG island poor regions are not subjected to evolutionary divergence. Moreover, due to the lack of significant differences in the number of repetitions of SSR motifs between gene sequences of the different plant species and lack of differences within the repetitive SSR motifs between gene sequences within species, the phylogenetic analysis did not show a clear and defined phylogenetic relationship. Therefore, further analysis of CpG islands and their convergence into TSSs of genes and involvement in evolutionary divergence will pave the way for a greater understanding of their roles in gene expression and gene evolution.

## Conclusion

The major aim of this work was to explore regulatory elements that can determine the expression of glucan endo-1,3-beta-glucosidase genes of *Solanum tuberosum* cultivar DM 1-3 516 R44. Consequently, the study showed transcription factors that serve as receptors, activators, and/or repressors of glucan endo-1,3-beta-glucosidase gene. In addition, transcription start sites, promoter regions, SSR motifs, and CpG islands in glucan endo-1,3-beta-glucosidase gene that plays role in the process of gene expression regulation were identified. The phylogenetic analysis revealed that the clustering patterns of the gene sequences were not entirely based on taxa. In general, this in silico analysis would allow for the understanding of regulatory mechanisms involved in glucan endo-1,3-beta-glucosidase gene expression and helps to identify gene regulatory elements in the promoter regions.

## Supplementary Information

**Additional file 1: Supplementary table 1** SSR motif occurrences by gene sequences

**Additional file 2: Supplementary table 2** List of the glucan endo-1,3-beta -glucosidase gene sequences from different plant species

**Additional file 3: Supplementary table 3** Genetic distance matrix

**Additional file 4: Supplementary table 4** Data matrix of the multiple sequence alignment

## Data Availability

The qualitative and quantitative data of this manuscript are available through the first author.

## Abbreviations

TSS: Transcription start site
MβGII: Motif of beta-glucosidase
TFs: Transcription factors
SSR: Simple sequence repeat
MEME: Multiple em for motif elicitation
NCBI: National center for biotechnology information
bp: Base pair
NNPP: Neural network promoter prediction
