# Supplementary material for: In silico analysis of promoter region and regulatory elements of glucan endo-1,3-beta-glucosidase encoding genes in Solanum tuberosum: cultivar DM 1-3 516 R44
Source: J Genet Eng Biotechnol. 2021 Sep 30;19:145. doi: 10.1186/s43141-021-00240-0 (PMC8484425; doi:10.1186/s43141-021-00240-0)
Supplement: Supplementary file 1 — Additional file 1: Supplementary table 1 SSR motif occurrences by gene sequences [file 43141_2021_240_MOESM1_ESM.docx]

Supplementary table 1 SSR motif occurrences by gene sequences

| **Gene ID** | **Motif length group** | **Types of SSR motifs found in the gene sequences, in two and above repeat number** | **Number of each type of motifs** |
| --- | --- | --- | --- |
| ID: 102588651 | Dimer | ag, at, ca, ct, ga, gt,ta,tc,tg | 9 |
|  | Trimer | aag, aca, acc, act, aga, agg, cct, ctc, ctt, gta,gtg,gtt, tac,tag, tca,tcc, tct, tga, tgc, tgg, tgt, tta,ttc, ttg | 24 |
|  | Tetramer | aaat, aatt, actt, attc,attt, ctcc,gtaa,tatt, tttc, tttg | 10 |
|  | Pentamer | ggggt | 1 |
|  | Hexamer | ccaaat | 1 |
| **Total** | | | **45** |
| ID: 102594958 | Dimer | ac, ag, at, ca, cg, ct, ga, gc, gt, ta, tc, tg | 12 |
|  | Trimer | aac, aag,aat,aca, acc, agg, agt, ata, atg, att, caa, cag,cat, cca, cga, cta, ctt, gaa, gcc,ggt, gta, gtg, gtt, taa, tac tag,tat,tcc, tct, tga, tgc,tgg,tgt,tcc, ttg | 35 |
|  | Tetramer | accc,agaa,attc,attt,cctc,cgat, ctga, ggtt, gtgg, taga, tcat, ttcg | 12 |
|  | Pentamer | aaaat, aataa, | 2 |
|  | Hexamer | tactat, tttgtt | 2 |
| **Total** | | | **63** |

**Supplementary table 1** **(*Continued*)**

| ID: 102601393 | Dimer | ac, ag, at, ca, cg, ct, gc, gt, ta, tc,tg | 11 |
| --- | --- | --- | --- |
|  | Trimer | aac,aat,acg,act,agc,agg,agt,ata, atg,att,caa, cta,ctc,gca,ggt,taa, tac,tat, tca,tct,tga,tgc,tgg,tgt,tta,ttc, | 26 |
|  | Tetramer | atta,catc,gaac,tatt,tgaa, | 5 |
|  | Pentamer | aaatc, tacgc, tcaaa | 3 |
|  | Hexamer | ccattt, tggagg | 2 |
| **Total** | | | **47** |
| ID: 102595473 | Dimer | ac, ag, at,ca,cg, ct,ga, gc,gt,ta,ca cg, ct,ga,gc,gt,ta,tc,tg | 19 |
|  | Trimer | aag,aat,aca, agg,ata,att, ctg, ctt, gaa,tag, tat, tct, tga, tgt, tta, ttc, ttg, | 17 |
|  | Tetramer | ataa,atag,caag, tcta, ttga,ttgt,tttg | 7 |
|  | Pentamer | atgat,gaaat | 2 |
|  | Hexamer | ----------- | 0 |
| **Total** | | | **45** |
| ID: 102593331 | Dimer | ac,ag,at,ca, cg,ct,ga,gc, gt,ta,tc,tg | 12 |
|  | Trimer | aat, aca ,acc,agg,agt, ata , att, caa,cat,cca, cta,ctc,ctg,ctt,gta, gtg, gtt, taa, tag, tat, tcc, tct, tga,tgc,tgg,tgt,ttc | 27 |
|  | Tetramer | aagt, aata,atga,caac,caat,ctcg, cttt,gaaa,gctg,ggcc,tatt, tgat,ttca, ttct, ttta, | 15 |
|  | Pentamer | aaaat, aattt, ataga, atcta, cttta, ggtgg | 6 |
|  | Hexamer | aattcc,catttt, ggggta, ttttct | 4 |
| **Total** | | | **64** |
| ID: 102578898 | Dimer | ac,ag,at,ca,ct,ga, at,gc,gt,ta,tc,tg, | 12 |
|  | Trimer | aac, aca, act, agg, agt, ata, att, caa, cct, cga, ctg, ctt, gaa, gag, gat, gct, gga, ggt, gta, gtg,taa,tac,tat, tca tct, tga, tgc, tgg, tgt,tta,ttc,ttg | 32 |
|  | Tetramer | aaca, agac, agca,agcc,attc, caaa, catt, ctaa, ggca,tgat, tgta tgtt, ttct, ttgc | 14 |
|  | Pentamer | aatct, ccaga, tcatt, tgcaa, tgctt | 5 |
|  | Hexamer | atgttt, taatag, tgagct | 3 |
| **Total** | | | **66** |

**Supplementary table 1** **(*Continued*)**

| ID: 1025835931 | | Dimer | | ac,ag, at, ca, ct,ga,gt,ta,tc,tg | 10 | | |
| --- | --- | --- | --- | --- | --- | --- | --- |
|  |  | Trimer | | aat, aca, act, aga, agc,atg, caa, cac,cct, cga,cta, ctc, ctg,ctt, gaa, gcc, tct,tga,tgg | 19 | | |
|  |  | Tetramer | | acaa, cgga, ctta,tctg | 4 | | |
|  |  | Pentamer | | tgcaa | 1 | | |
|  |  | Hexamer | | cccaat | 1 | | |
| **Total** | | | | | **35** | | |
| ID: 102595860 | | Dimer | | ac, ag, at, ca, cg, ct, ga, gt, ta, tc, tg | 11 | | |
|  |  | Trimer | | aac,aag,aat, aga,agc,agg, atg, att, caa, cag,cat,cca,ctg,ctt, gag, gga, gtg,tac, tca,tga, tgc, tgg, ttg, | 23 | | |
|  |  | Tetramer | | gaac | 1 | | |
|  |  | Pentamer | | gaaat | 1 | | |
|  |  | Hexamer | | -------------- | 0 | | |
| Total | | | | | **36** | | |
| D:102605560 | Dimer | | ac, ag, at, ca,ct, ga, gt, ta, tc,tg | | | 10 |  |
|  | Trimer | | aac, aag, aca, acc,aga, agc,agg, ata, atc, atg,att, caa, cag, cat, cca, ctg, ctt, gaa, gat, gca, gct, gga, ggt, gta, gtg, gtt, taa, tac, tag,tat, tcc, tcg, tct, tga,tgc, tgg, tgt, tta, ttc, ttg | | | 40 |  |
|  | Tetramer | | aaat, aagt, agaa, agac, agtt, atga,attt, caat, gata, gtaa, tcaa, tgat, ttac,ttta, tttc | | | 15 |  |
|  | Pentamer | | aataa, agtgt, agttt,atatg,gaagt, gggat, gtttt,tctat, ttata, ttatg, tttta | | | 11 |  |
|  | Hexamer | | cctctg, cttttc | | | 2 |  |
| **Total** | | | | | | **78** |  |
| ID: 102601178 | Dimer | | ac. ag, at, ca, cg, ct, ga, gc,gt, ta, tc,tg | | | 12 |  |
|  | Trimer | | aag, aat, agc, agg, cgt,ctc, ctt,gaa, gag, gat,gcg, gga, ggc, ggt,gtg, gtt, taa, tac, tat, tct , tgc,tta,ttc | | | 23 |  |
|  | Tetramer | | aaag, aagt, ctat,tatt,ttca, ttct | | | 6 |  |
|  | Pentamer | | tttta | | | 1 |  |
|  | Hexamer | | -------------- | | | 0 |  |
| **Total** | | | | | | **42** |  |

**Supplementary table 1** **(*Continued*)**

| ID:102587248 | Dimer | | ac, ag, at, ca, cg, ct, ga, gc,gt, ta , tc,tg | | 12 |
| --- | --- | --- | --- | --- | --- |
|  | Trimer | | aac, aag, aat, aca, acc, act, agt, ata, atc, atg, att, caa, cac,cat, cct, cta, ctc, ctt, gat, gca, gcg, gct, gga, taa, tag, tat ,tca, tcc, tct ,tgc, tgg, tgt, tta, ttc, ttg | | 35 |
|  | Tetramer | | aaag, acaa, agat, attt,cttt, gaat, gata,ggag, gggt, ggtg, gttg, tctt, tgat, tggg, ttgg, tttc | | 16 |
|  | Pentamer | | gtatt, gtcta, tattg, tgggg | | 4 |
|  | Hexamer | | tgggg, tgttat,ttggaa, ttttag, ttttct | | 5 |
| **Total** | | | | | **72** |
| ID: 102604922 | Dimer | | ag, at, ca,cg, ct, ga, ta, tc, tg | | 9 |
|  | Trimer | | aat, aca, agg, ata.cat,cga, ctg,ctt,gaa, gtt, taa, tac, tag, tca, tct, tgt | | 16 |
|  | Tetramer | | agca,caac,ctag, gcag, ggag,tgaa | | 6 |
|  | Pentamer | | aatcg, caatg , caatg | | 3 |
|  | Hexamer | | --------------- | | 0 |
| **Total** | | | | | **34** |
| ID: 102605428 | | Dimer | | ac, ag,at, ca , ct, ga,gc, gt, ta, tc, tg | 11 |
|  |  | Trimer | | aac,aat,act, agt, atc, cag, cta, ctc, gaa,gag,gat, gct, gtt,taa, tac, tat, tgc,tgg,tgt, ttc,ttg | 21 |
|  |  | Tetramer | | aaag, acct, aggg, attt, gatt, tcta, tctt, tgta, tttc | 9 |
|  |  | Pentamer | | atgtt, ttgac | 2 |
|  |  | Hexamer | | --------------- | 0 |
| **Total** | | | | | **43** |
| ID: 102596927 | | Dimer | | ac, ag, at, ca, cg, ct, ga, gc, gt,ta, tc, tg | 12 |
|  |  | Trimer | | aag, aat, aga, agg, ata, atg,att, cac,cat,cca,cga,ctc, ctt, gaa, gga, gta, gtc,tat, tgt, tta, ttg | 21 |
|  |  | Tetramer | | aagt, agat, caat, cata, cctc,taaa, tcct, ttcc | 8 |
|  |  | Pentamer | | aaggg, atcga, atttt, gtttt | 4 |
|  |  | Hexamer | | cttcca, tagaag, ttgttt | 3 |
| **Total** | | | | | **48** |
| ID: 102583800 | | Dimer | | ac, ag, at, ca, ct, ga, gc, gt, ta, tc, tg | 11 |
|  |  | Trimer | | aac, aat,aca,acc, acg,act, agc, agt, ata, att,cat, ccg, cga, cgc, cgg, cgt, cta, ctc, ctg, ctt,gct, ggt, tat, tca, tcc, tcg, tga, tgt, tta, ttc, ttg | 31 |
|  |  | Tetramer | | agtg, gtag, tcat, ttag, ttca | 5 |
|  |  | Pentamer | | ctttg | 1 |
|  |  | Hexamer | | cgacgc, ttagtt | 2 |
| **Total** | | | | | **50** |

**Supplementary table 1** **(*Continued*)**

| ID: 102581946 | | | Dimer | | ac, ag, at, ca, cg, ct, ga, gc, gt, ta, tc,tg | 12 | | | | |
| --- | --- | --- | --- | --- | --- | --- | --- | --- | --- | --- |
|  |  |  | Trimer | | aag, aat, aca,aga, agt,ata, atg, att,caa, cac, cat, cta, ctg, ctt, gaa, gca, gga,gta, gtg, gtt, tac, tat, tca, tct,tga, tgc, tgt, tta, ttc,ttg | 30 | | | | |
|  |  |  | Tetramer | | aaca,aact, attg,catg,cgaa,ctag,gaag,gata,gatg,gtag, gtca,tgta, ttca, ttta, tttc | 15 | | | | |
|  |  |  | Pentamer | | aacta,ggtaa, ttttc | 3 | | | | |
|  |  |  | Hexamer | | cttgca,gggtaa | 2 | | | | |
| **Total** | | | | | | **62** | | |  |  |
| ID: 102578810 | | | Dimer | | ac,ag, at, ca, cg,ct,ga, gc, gt,ta,tc,tg | | | | 12 |  |
|  |  |  | Trimer | | aac, aag, aat, aca, agc, ata, atc, att,caa, cac, cag, cgc, cgg, cgt,ctc, ctt,gaa,gac,ggt,gta,gtg,gtt,taa, tat,tca, tcc,tct,tgc, tgg,tta,ttc,ttg | | | | 32 |  |
|  |  |  | Tetramer | | agct, agta, atct, atgt, caaa, cact,cagg,ctgg, gatg, gttt,tatt, tgag,ttaa,ttat,ttta, tttg | | | | 16 |  |
|  |  |  | Pentamer | | tccga, tttat | | | | 2 |  |
|  |  |  | Hexamer | | caaaat, tttccc | | | | 2 |  |
| **Total** | | | | | | | | | **64** |  |
| ID: 102595638 | | | Dimer | | ac,ag,at,ca,cg, ct, ga, gt,ta,tc, tg | | | | 11 |  |
|  |  |  | Trimer | | aac, aag, aca, act, agg,ata,atc,att,caa,cat,cta,ctc,ctt,gag,gct,gtg,gtt,taa,tac,tat,tca,tct,tga,tgc,tgg,tgt,tta,ttc,ttg | | | | 29 |  |
|  |  |  | Tetramer | | acat,actt, agga,atta, attt,cata,cttt, gaag,gtat, gtga,gtta, gttt,ttca, ttcc, ttta, tttg | | | | 16 |  |
|  |  |  | Pentamer | | aaagt, agtat,ctttt,tatgt,tcttt,tttaa | | | | 6 |  |
|  |  |  | Hexamer | | gcactg,ttggac,tttaat,tttttg | | | | 4 |  |
| **Total** | | | | | | | | | **66** |  |
| ID: 102589208 | | | Dimer | | ac, ag, at, ca, cg, ct, ga ,gc, gt, ta, tc, tg | | | | 12 |  |
|  |  |  | Trimer | | aat, agg,ata, atc, att, caa, cag, cta, gaa, gct, gga, gta,taa, tat, tct, tga,tta, ttg | | | | 18 |  |
|  |  |  | Tetramer | | aacc, catg, taat, taca, tctt, ttaa, ttat, ttta | | | | 8 |  |
|  |  |  | Pentamer | | aaatc, ttata | | | | 2 |  |
|  |  |  | Hexamer | | atcaac | | | | 1 |  |
| **Total** | | | | | | | | | **41** |  |
