# Supplementary material for: In silico analysis of promoter region and regulatory elements of glucan endo-1,3-beta-glucosidase encoding genes in Solanum tuberosum: cultivar DM 1-3 516 R44
Source: J Genet Eng Biotechnol. 2021 Sep 30;19:145. doi: 10.1186/s43141-021-00240-0 (PMC8484425; doi:10.1186/s43141-021-00240-0)
Supplement: Supplementary file 2 — Additional file 2: Supplementary table 2 List of the glucan endo-1,3-beta -glucosidase gene sequences from different plant species [file 43141_2021_240_MOESM2_ESM.docx]

Supplementary table 2 List of the glucan endo-1,3-beta -glucosidase gene sequences from different plant species

| **S no** Gene ID Species |
| --- |
| 1. ID102588651_*Solanum tuberosum* |
| 1. ID102594958_*Solanum tuberosum* |
| 1. ID102601393_*Solanum tuberosum* |
| 1. ID102595473_*Solanum tuberosum* |
| 1. ID102593331_*Solanum tuberosum* |
| 1. ID102578898_*Solanum tuberosum* |
| 1. ID102583593_*Solanum tuberosum* |
| 1. ID102605560_*Solanum tuberosum* |
| 1. ID102601178_*Solanum tuberosum* |
| 1. ID102587248_*Solanum tuberosum* |
| 1. ID102604922_*Solanum tuberosum* |
| 1. ID102605428_*Solanum tuberosum* |
| 1. ID102596927_*Solanum tuberosum* |
| 1. ID102595860_*Solanum tuberosum* |
| 1. ID102583800_*Solanum tuberosum* |
| 1. ID102581946_*Solanum tuberosum* |
| 1. ID102578810_*Solanum tuberosum* |
| 1. ID102595638_*Solanum tuberosum* |
| 1. ID102589208_*Solanum tuberosum* |
| 1. ID107823411_*Nicotiana tabacum* |
| 1. ID107825406_*Nicotiana tabacum* |
| 1. ID107789548_*Nicotiana tabacum* |
| 1. ID107763655_*Nicotiana tabacum* |
| 1. ID107801151_*Nicotiana tabacum* |
| 1. ID107777766_*Nicotiana tabacum* |
| 1. ID107814850_*Nicotiana tabacum* |
| 1. ID107763289_*Nicotiana tabacum* |
| 1. ID107784423_*Nicotiana tabacum* |
| 1. ID107820469_*Nicotiana tabacum* |
| 1. ID107803828_*Nicotiana tabacum* |
| 1. ID107824944_*Nicotiana tabacum* |
| 1. ID543987_*Solanum lycopersicum* |
| 1. ID543986_*Solanum lycopersicum* |
| 1. ID101245933_*Solanum lycopersicum* |
| 1. ID824893_*Arabidopsis thaliana* |
| 1. ID834215_*Arabidopsis thaliana* |
| 1. ID824891_*Arabidopsis thaliana* |
| 1. ID832156_*Arabidopsis thaliana* |
| 1. ID824894_*Arabidopsis thaliana* |
| 1. ID832155_*Arabidopsis thaliana* |
