# Supplementary material for: In silico analysis of promoter region and regulatory elements of glucan endo-1,3-beta-glucosidase encoding genes in Solanum tuberosum: cultivar DM 1-3 516 R44
Source: J Genet Eng Biotechnol. 2021 Sep 30;19:145. doi: 10.1186/s43141-021-00240-0 (PMC8484425; doi:10.1186/s43141-021-00240-0)
Supplement: Supplementary file 3 — Additional file 3: Supplementary table 3 Genetic distance matrix [file 43141_2021_240_MOESM3_ESM.docx]

Supplementary table 3 Genetic distance matrix

|  | 1 | 2 | 3 | 4 | 5 | 6 | 7 | 8 | 9 | 10 | 11 | 12 | 13 | 14 | 15 |
| --- | --- | --- | --- | --- | --- | --- | --- | --- | --- | --- | --- | --- | --- | --- | --- |
| 1 |  |  |  |  |  |  |  |  |  |  |  |  |  |  |  |
| 2 | 0.748 |  |  |  |  |  |  |  |  |  |  |  |  |  |  |
| 3 | 0.720 | 0.733 |  |  |  |  |  |  |  |  |  |  |  |  |  |
| 4 | 0.737 | 0.744 | 0.717 |  |  |  |  |  |  |  |  |  |  |  |  |
| 5 | 0.728 | 0.725 | 0.737 | 0.718 |  |  |  |  |  |  |  |  |  |  |  |
| 6 | 0.708 | 0.719 | 0.730 | 0.704 | 0.721 |  |  |  |  |  |  |  |  |  |  |
| 7 | 0.732 | 0.734 | 0.756 | 0.711 | 0.742 | 0.747 |  |  |  |  |  |  |  |  |  |
| 8 | 0.717 | 0.728 | 0.711 | 0.734 | 0.710 | 0.715 | 0.756 |  |  |  |  |  |  |  |  |
| 9 | 0.732 | 0.733 | 0.745 | 0.711 | 0.727 | 0.736 | 0.746 | 0.738 |  |  |  |  |  |  |  |
| 10 | 0.764 | 0.719 | 0.739 | 0.723 | 0.743 | 0.722 | 0.734 | 0.740 | 0.728 |  |  |  |  |  |  |
| 11 | 0.737 | 0.733 | 0.731 | 0.728 | 0.732 | 0.719 | 0.739 | 0.725 | 0.719 | 0.739 |  |  |  |  |  |
| 12 | 0.725 | 0.737 | 0.728 | 0.722 | 0.743 | 0.726 | 0.740 | 0.750 | 0.742 | 0.736 | 0.747 |  |  |  |  |
| 13 | 0.730 | 0.743 | 0.730 | 0.738 | 0.736 | 0.726 | 0.753 | 0.722 | 0.720 | 0.730 | 0.721 | 0.729 |  |  |  |
| 14 | 0.744 | 0.724 | 0.720 | 0.740 | 0.725 | 0.722 | 0.721 | 0.707 | 0.739 | 0.739 | 0.737 | 0.746 | 0.742 |  |  |
| 15 | 0.729 | 0.730 | 0.748 | 0.731 | 0.745 | 0.720 | 0.748 | 0.743 | 0.731 | 0.753 | 0.734 | 0.738 | 0.731 | 0.738 |  |
| 16 | 0.736 | 0.721 | 0.737 | 0.713 | 0.718 | 0.731 | 0.725 | 0.723 | 0.726 | 0.733 | 0.726 | 0.756 | 0.740 | 0.728 | 0.750 |
| 17 | 0.733 | 0.734 | 0.754 | 0.737 | 0.735 | 0.742 | 0.769 | 0.745 | 0.733 | 0.736 | 0.732 | 0.685 | 0.734 | 0.732 | 0.723 |
| 18 | 0.738 | 0.730 | 0.725 | 0.699 | 0.717 | 0.711 | 0.726 | 0.740 | 0.715 | 0.711 | 0.719 | 0.744 | 0.716 | 0.724 | 0.723 |
| 19 | 0.711 | 0.726 | 0.721 | 0.710 | 0.718 | 0.725 | 0.730 | 0.740 | 0.721 | 0.718 | 0.716 | 0.705 | 0.723 | 0.740 | 0.750 |
| 20 | 0.723 | 0.737 | 0.736 | 0.716 | 0.721 | 0.722 | 0.739 | 0.730 | 0.726 | 0.718 | 0.732 | 0.732 | 0.737 | 0.743 | 0.745 |
| 21 | 0.717 | 0.732 | 0.717 | 0.740 | 0.728 | 0.714 | 0.750 | 0.728 | 0.703 | 0.740 | 0.724 | 0.738 | 0.729 | 0.740 | 0.746 |
| 22 | 0.736 | 0.729 | 0.719 | 0.731 | 0.733 | 0.719 | 0.740 | 0.738 | 0.718 | 0.736 | 0.713 | 0.743 | 0.736 | 0.716 | 0.734 |
| 23 | 0.751 | 0.756 | 0.731 | 0.730 | 0.718 | 0.741 | 0.753 | 0.729 | 0.715 | 0.728 | 0.731 | 0.746 | 0.731 | 0.741 | 0.728 |
| 24 | 0.720 | 0.744 | 0.714 | 0.720 | 0.729 | 0.719 | 0.741 | 0.724 | 0.723 | 0.741 | 0.730 | 0.736 | 0.728 | 0.736 | 0.750 |
| 25 | 0.729 | 0.742 | 0.726 | 0.716 | 0.719 | 0.709 | 0.741 | 0.736 | 0.721 | 0.728 | 0.724 | 0.738 | 0.710 | 0.735 | 0.736 |
| 26 | 0.717 | 0.740 | 0.720 | 0.737 | 0.727 | 0.758 | 0.738 | 0.731 | 0.728 | 0.749 | 0.744 | 0.745 | 0.735 | 0.747 | 0.740 |
| 27 | 0.734 | 0.748 | 0.692 | 0.693 | 0.736 | 0.727 | 0.725 | 0.745 | 0.731 | 0.741 | 0.740 | 0.736 | 0.724 | 0.747 | 0.766 |
| 28 | 0.735 | 0.728 | 0.707 | 0.742 | 0.737 | 0.736 | 0.754 | 0.737 | 0.717 | 0.743 | 0.731 | 0.756 | 0.736 | 0.709 | 0.733 |
| 29 | 0.739 | 0.734 | 0.712 | 0.717 | 0.728 | 0.699 | 0.739 | 0.735 | 0.715 | 0.744 | 0.749 | 0.749 | 0.713 | 0.722 | 0.750 |
| 30 | 0.750 | 0.735 | 0.744 | 0.719 | 0.708 | 0.724 | 0.726 | 0.738 | 0.728 | 0.722 | 0.744 | 0.768 | 0.730 | 0.733 | 0.750 |
| 31 | 0.746 | 0.743 | 0.759 | 0.708 | 0.753 | 0.716 | 0.741 | 0.750 | 0.753 | 0.723 | 0.736 | 0.768 | 0.723 | 0.748 | 0.733 |
| 32 | 0.725 | 0.729 | 0.729 | 0.696 | 0.722 | 0.715 | 0.748 | 0.732 | 0.723 | 0.723 | 0.723 | 0.729 | 0.736 | 0.734 | 0.728 |
| 33 | 0.709 | 0.722 | 0.727 | 0.716 | 0.720 | 0.712 | 0.733 | 0.726 | 0.707 | 0.698 | 0.727 | 0.736 | 0.737 | 0.730 | 0.734 |
| 34 | 0.725 | 0.719 | 0.735 | 0.712 | 0.713 | 0.724 | 0.704 | 0.744 | 0.727 | 0.740 | 0.724 | 0.720 | 0.726 | 0.729 | 0.741 |
| 35 | 0.731 | 0.760 | 0.742 | 0.755 | 0.749 | 0.749 | 0.764 | 0.736 | 0.742 | 0.744 | 0.745 | 0.741 | 0.760 | 0.735 | 0.745 |
| 36 | 0.743 | 0.729 | 0.745 | 0.742 | 0.727 | 0.750 | 0.717 | 0.750 | 0.739 | 0.737 | 0.757 | 0.760 | 0.749 | 0.736 | 0.729 |
| 37 | 0.714 | 0.756 | 0.750 | 0.735 | 0.747 | 0.747 | 0.736 | 0.740 | 0.750 | 0.741 | 0.739 | 0.740 | 0.732 | 0.734 | 0.747 |
| 38 | 0.761 | 0.719 | 0.728 | 0.749 | 0.722 | 0.741 | 0.730 | 0.763 | 0.739 | 0.733 | 0.751 | 0.758 | 0.730 | 0.756 | 0.757 |
| 39 | 0.721 | 0.726 | 0.719 | 0.742 | 0.724 | 0.734 | 0.734 | 0.710 | 0.735 | 0.738 | 0.726 | 0.721 | 0.728 | 0.728 | 0.729 |
| 40 | 0.745 | 0.745 | 0.761 | 0.739 | 0.723 | 0.738 | 0.734 | 0.766 | 0.748 | 0.749 | 0.762 | 0.727 | 0.744 | 0.753 | 0.738 |

**Supplementary table 3** **(*continued*)**

|  | 16 | 17 | 18 | 19 | 20 | 21 | 22 | 23 | 24 | 25 | 26 | 27 | 28 | 29 |
| --- | --- | --- | --- | --- | --- | --- | --- | --- | --- | --- | --- | --- | --- | --- |
|  |  |  |  |  |  |  |  |  |  |  |  |  |  |  |
| 17 | 0.735 |  |  |  |  |  |  |  |  |  |  |  |  |  |
| 18 | 0.716 | 0.714 |  |  |  |  |  |  |  |  |  |  |  |  |
| 19 | 0.716 | 0.742 | 0.715 |  |  |  |  |  |  |  |  |  |  |  |
| 20 | 0.734 | 0.729 | 0.721 | 0.708 |  |  |  |  |  |  |  |  |  |  |
| 21 | 0.729 | 0.732 | 0.725 | 0.708 | 0.733 |  |  |  |  |  |  |  |  |  |
| 22 | 0.734 | 0.732 | 0.715 | 0.705 | 0.720 | 0.725 |  |  |  |  |  |  |  |  |
| 23 | 0.721 | 0.719 | 0.706 | 0.723 | 0.728 | 0.710 | 0.740 |  |  |  |  |  |  |  |
| 24 | 0.740 | 0.739 | 0.719 | 0.711 | 0.709 | 0.722 | 0.709 | 0.729 |  |  |  |  |  |  |
| 25 | 0.728 | 0.732 | 0.710 | 0.750 | 0.720 | 0.731 | 0.729 | 0.704 | 0.719 |  |  |  |  |  |
| 26 | 0.725 | 0.745 | 0.747 | 0.733 | 0.729 | 0.738 | 0.733 | 0.737 | 0.742 | 0.735 |  |  |  |  |
| 27 | 0.728 | 0.741 | 0.721 | 0.724 | 0.716 | 0.756 | 0.718 | 0.722 | 0.733 | 0.721 | 0.739 |  |  |  |
| 28 | 0.750 | 0.748 | 0.713 | 0.734 | 0.748 | 0.709 | 0.747 | 0.736 | 0.749 | 0.729 | 0.721 | 0.752 |  |  |
| 29 | 0.734 | 0.712 | 0.687 | 0.748 | 0.704 | 0.728 | 0.715 | 0.719 | 0.707 | 0.708 | 0.722 | 0.721 | 0.722 |  |
| 30 | 0.730 | 0.738 | 0.722 | 0.758 | 0.739 | 0.734 | 0.755 | 0.725 | 0.733 | 0.728 | 0.763 | 0.737 | 0.745 | 0.737 |
| 31 | 0.742 | 0.762 | 0.746 | 0.749 | 0.724 | 0.729 | 0.762 | 0.727 | 0.736 | 0.727 | 0.702 | 0.741 | 0.756 | 0.745 |
| 32 | 0.726 | 0.723 | 0.696 | 0.711 | 0.714 | 0.729 | 0.715 | 0.734 | 0.708 | 0.744 | 0.735 | 0.701 | 0.733 | 0.724 |
| 33 | 0.739 | 0.722 | 0.727 | 0.735 | 0.742 | 0.721 | 0.713 | 0.719 | 0.723 | 0.724 | 0.732 | 0.706 | 0.726 | 0.717 |
| 34 | 0.721 | 0.708 | 0.723 | 0.711 | 0.710 | 0.733 | 0.716 | 0.724 | 0.728 | 0.721 | 0.750 | 0.740 | 0.730 | 0.706 |
| 35 | 0.733 | 0.752 | 0.736 | 0.722 | 0.761 | 0.738 | 0.738 | 0.735 | 0.735 | 0.742 | 0.752 | 0.743 | 0.735 | 0.740 |
| 36 | 0.764 | 0.747 | 0.735 | 0.749 | 0.747 | 0.742 | 0.732 | 0.754 | 0.742 | 0.736 | 0.766 | 0.744 | 0.742 | 0.770 |
| 37 | 0.720 | 0.752 | 0.718 | 0.738 | 0.740 | 0.731 | 0.736 | 0.764 | 0.749 | 0.738 | 0.717 | 0.741 | 0.733 | 0.762 |
| 38 | 0.770 | 0.753 | 0.761 | 0.747 | 0.744 | 0.745 | 0.740 | 0.746 | 0.744 | 0.732 | 0.747 | 0.739 | 0.762 | 0.753 |
| 39 | 0.736 | 0.727 | 0.720 | 0.719 | 0.732 | 0.717 | 0.728 | 0.704 | 0.730 | 0.731 | 0.745 | 0.732 | 0.726 | 0.704 |
| 40 | 0.741 | 0.741 | 0.725 | 0.731 | 0.731 | 0.735 | 0.728 | 0.749 | 0.734 | 0.752 | 0.752 | 0.751 | 0.746 | 0.728 |

**Supplementary table 3**  **(*continued*)**

|  | 30 | 31 | 32 | 33 | 34 | 35 | 36 | 37 | 38 | 39 | 40 |
| --- | --- | --- | --- | --- | --- | --- | --- | --- | --- | --- | --- |
|  |  |  |  |  |  |  |  |  |  |  |  |
| 31 | 0.713 |  |  |  |  |  |  |  |  |  |  |
| 32 | 0.729 | 0.746 |  |  |  |  |  |  |  |  |  |
| 33 | 0.736 | 0.709 | 0.733 |  |  |  |  |  |  |  |  |
| 34 | 0.713 | 0.754 | 0.738 | 0.719 |  |  |  |  |  |  |  |
| 35 | 0.752 | 0.750 | 0.736 | 0.764 | 0.743 |  |  |  |  |  |  |
| 36 | 0.731 | 0.733 | 0.757 | 0.748 | 0.724 | 0.751 |  |  |  |  |  |
| 37 | 0.758 | 0.745 | 0.748 | 0.759 | 0.739 | 0.734 | 0.733 |  |  |  |  |
| 38 | 0.761 | 0.715 | 0.727 | 0.729 | 0.744 | 0.754 | 0.730 | 0.745 |  |  |  |
| 39 | 0.731 | 0.743 | 0.728 | 0.735 | 0.718 | 0.749 | 0.750 | 0.751 | 0.747 |  |  |
| 40 | 0.727 | 0.744 | 0.738 | 0.743 | 0.737 | 0.736 | 0.748 | 0.751 | 0.754 | 0.747 |  |
