# Supplementary material for: In silico analysis of promoter region and regulatory elements of glucan endo-1,3-beta-glucosidase encoding genes in Solanum tuberosum: cultivar DM 1-3 516 R44
Source: J Genet Eng Biotechnol. 2021 Sep 30;19:145. doi: 10.1186/s43141-021-00240-0 (PMC8484425; doi:10.1186/s43141-021-00240-0)
Supplement: Supplementary file 4 — Additional file 4: Supplementary table 4 Data matrix of the multiple sequence alignment [file 43141_2021_240_MOESM4_ESM.docx]

**Supplementary table 4** Data matrix of the multiple sequence alignment

| ID102593331 | | 100 | |  | |  | |  | |  | |  | | |  | |  | |  | |  | |  | |  | |  | |  | |  | |  | | |  | |  |  |
| --- | --- | --- | --- | --- | --- | --- | --- | --- | --- | --- | --- | --- | --- | --- | --- | --- | --- | --- | --- | --- | --- | --- | --- | --- | --- | --- | --- | --- | --- | --- | --- | --- | --- | --- | --- | --- | --- | --- | --- |
| ID101245933 | | 84.8 | | 100 | |  | |  | |  | |  | | |  | |  | |  | |  | |  | |  | |  | |  | |  | |  | | |  | |  |  |
| ID102595638 | | 38.1 | | 37.7 | | 100 | |  | |  | |  | | |  | |  | |  | |  | |  | |  | |  | |  | |  | |  | | |  | |  |  |
| ID102601178 | | 39.9 | | 40.2 | | 41.7 | | 100 | |  | |  | | |  | |  | |  | |  | |  | |  | |  | |  | |  | |  | | |  | |  |  |
| ID102594958 | | 34.7 | | 35 | | 36.9 | | 38.5 | | 100 | |  | | |  | |  | |  | |  | |  | |  | |  | |  | |  | |  | | |  | |  |  |
| ID102604922 | | 36.7 | | 37.6 | | 40.7 | | 43.6 | | 46.6 | | 100 | | |  | |  | |  | |  | |  | |  | |  | |  | |  | |  | | |  | |  |  |
| ID834215 | | 39.1 | | 39.2 | | 37.4 | | 40 | | 46.9 | | 49.3 | | | 100 | |  | |  | |  | |  | |  | |  | |  | |  | |  | | |  | |  |  |
| ID102583593 | | 38.6 | | 38.1 | | 38.1 | | 36.3 | | 49.3 | | 54 | | | 59.1 | | 100 | |  | |  | |  | |  | |  | |  | |  | |  | | |  | |  |  |
| ID102583800 | | 36.6 | | 37.3 | | 38.2 | | 36.9 | | 48.4 | | 49.5 | | | 53.8 | | 69.8 | | 100 | |  | |  | |  | |  | |  | |  | |  | | |  | |  |  |
| ID832156 | | 32.8 | | 31.5 | | 31.7 | | 40.6 | | 31.9 | | 30.4 | | | 31.2 | | 27.6 | | 28.2 | | 100 | |  | |  | |  | |  | |  | |  | | |  | |  |  |
| ID832155 | | 31.1 | | 31 | | 30.5 | | 37.1 | | 29.4 | | 31.4 | | | 28.9 | | 23.8 | | 26.7 | | 88.2 | | 100 | |  | |  | |  | |  | |  | | |  | |  |  |
| ID102595473 | | 33.3 | | 32.7 | | 35.8 | | 39 | | 31.3 | | 39.8 | | | 31.7 | | 26.1 | | 27.7 | | 46.4 | | 45.2 | | 100 | |  | |  | |  | |  | | |  | |  |  |
| ID102589208 | | 35.2 | | 35.6 | | 36.6 | | 39.9 | | 34.8 | | 32.4 | | | 31.8 | | 25 | | 30.2 | | 46.3 | | 45 | | 60 | | 100 | |  | |  | |  | | |  | |  |  |
| ID543986 | | 34.9 | | 34.8 | | 36.7 | | 39 | | 33.6 | | 32.1 | | | 30.4 | | 25.1 | | 29.2 | | 46 | | 45.2 | | 58.9 | | 88.1 | | 100 | |  | |  | | |  | |  |  |
| ID107825406 | | 36.8 | | 35.5 | | 35.4 | | 38 | | 32.6 | | 31.3 | | | 30 | | 26.6 | | 29.6 | | 47.2 | | 45.1 | | 57.9 | | 65.7 | | 63.6 | | 100 | |  | | |  | |  |  |
| ID107789548 | | 35.5 | | 34.9 | | 35.8 | | 38.1 | | 33.6 | | 30.6 | | | 29.1 | | 26.6 | | 31.4 | | 46.5 | | 45 | | 60.3 | | 69.3 | | 66.7 | | 88.3 | | 100 | | |  | |  |  |
| ID107763289 | | 35.8 | | 34.7 | | 36.4 | | 39.7 | | 33.4 | | 31.6 | | | 28.6 | | 26.1 | | 31.9 | | 47.8 | | 48.3 | | 60.9 | | 72.4 | | 71 | | 86.1 | | 90 | | | 100 | |  |  |
| ID107763655 | | 35.6 | | 35.2 | | 34.2 | | 37 | | 33.1 | | 31 | | | 27.9 | | 27.6 | | 30.6 | | 46.6 | | 45.1 | | 59 | | 65.6 | | 62.9 | | 66.2 | | 69.2 | | | 69.9 | | 100 |  |
| ID107777766 | | 36.9 | | 34.7 | | 34.5 | | 37.8 | | 33.2 | | 31.2 | | | 29.5 | | 29.1 | | 30.4 | | 46.6 | | 46 | | 59.4 | | 64.1 | | 62 | | 64.8 | | 67.5 | | | 67.6 | | 82.7 | 100 |
| ID107823411 | | 33.1 | | 32.8 | | 34.1 | | 36.6 | | 30.1 | | 32.9 | | | 33.4 | | 31.5 | | 33.4 | | 47.5 | | 46.2 | | 48.9 | | 48.3 | | 47.8 | | 45.9 | | 47.8 | | | 49.5 | | 44.5 | 45.9 |
| ID107801151 | | 33.4 | | 33.6 | | 34.8 | | 37.4 | | 30.1 | | 33.5 | | | 33 | | 34 | | 34.1 | | 47.5 | | 45.9 | | 51.1 | | 49.5 | | 48.7 | | 47 | | 48.6 | | | 50.8 | | 44.9 | 46.1 |
| ID102605560 | | 32.8 | | 33.4 | | 34.6 | | 40.7 | | 30.6 | | 31.8 | | | 32.3 | | 33.5 | | 30.6 | | 47.6 | | 47.5 | | 51.3 | | 51.6 | | 51 | | 49.5 | | 49.1 | | | 50.8 | | 47.6 | 47.5 |
| ID543987 | | 34.3 | | 34.4 | | 35.2 | | 38.6 | | 30.7 | | 31.8 | | | 34.7 | | 33 | | 31.1 | | 47.4 | | 44.7 | | 50.7 | | 49.3 | | 48.1 | | 46.4 | | 47.4 | | | 49.4 | | 46 | 45.9 |
| ID102605428 | | 36.4 | | 35.1 | | 33.7 | | 42 | | 31.7 | | 32.6 | | | 32 | | 30.1 | | 30.4 | | 45.7 | | 46.5 | | 52 | | 51.8 | | 50.8 | | 51.1 | | 50.5 | | | 53.6 | | 49.5 | 50.5 |
| ID102596927 | | 36.9 | | 35.6 | | 34.2 | | 37.4 | | 31.5 | | 30.9 | | | 30.2 | | 31.5 | | 30 | | 46.1 | | 45.8 | | 46.8 | | 47.9 | | 47.9 | | 45.3 | | 46.8 | | | 48.9 | | 46.1 | 46.8 |
| ID107814850 | | 36.1 | | 35.1 | | 33.2 | | 37.1 | | 32.4 | | 31.3 | | | 30.5 | | 29.6 | | 27.7 | | 45.2 | | 44.5 | | 45.4 | | 47.6 | | 47.4 | | 44.9 | | 47.2 | | | 49.2 | | 46 | 45.9 |
| ID824894 | | 34.2 | | 31.3 | | 33.7 | | 36.9 | | 31.4 | | 30.6 | | | 30.1 | | 29.8 | | 28.7 | | 43.7 | | 42.6 | | 42.3 | | 41.9 | | 41 | | 40.3 | | 40.7 | | | 41.1 | | 39.7 | 41.1 |
| ID824893 | | 30.1 | | 26.5 | | 31.9 | | 36.7 | | 29 | | 27.7 | | | 27.7 | | 27.9 | | 27.7 | | 44.6 | | 42.9 | | 42.3 | | 43 | | 41.4 | | 41.2 | | 41.7 | | | 44.6 | | 40.7 | 42 |
| ID824891 | | 31.2 | | 27.8 | | 31.6 | | 37.3 | | 30 | | 29.8 | | | 28.4 | | 27.9 | | 27.7 | | 43.5 | | 40.7 | | 42.1 | | 41.5 | | 39.6 | | 41.1 | | 41.9 | | | 44.1 | | 40.2 | 42.8 |
| ID102601393 | | 29 | | 30.9 | | 33.8 | | 37.2 | | 31.1 | | 31.8 | | | 30.2 | | 28.3 | | 32.6 | | 36.4 | | 32.1 | | 36.6 | | 36.5 | | 32.6 | | 33.2 | | 32.9 | | | 35 | | 35.1 | 34.5 |
| ID102588651 | | 32.4 | | 33.2 | | 33.9 | | 38.7 | | 32.1 | | 32.5 | | | 32.4 | | 23.5 | | 27.9 | | 31.3 | | 31.3 | | 31.1 | | 32 | | 29.6 | | 29.1 | | 28.1 | | | 32 | | 30.4 | 29.5 |
| ID107784423 | | 34.8 | | 35.1 | | 38.6 | | 42 | | 33.2 | | 32.8 | | | 32.7 | | 32.3 | | 32.3 | | 46.8 | | 45.4 | | 50.4 | | 49.2 | | 47.4 | | 47.7 | | 47.4 | | | 50.5 | | 47.3 | 47.6 |
| ID102581946 | | 34.5 | | 34.8 | | 36.9 | | 41.5 | | 32.4 | | 34,63 | | | 31.1 | | 31.8 | | 33.9 | | 42.2 | | 39.9 | | 43.7 | | 42.9 | | 40.3 | | 41.8 | | 41.9 | | | 42.8 | | 41 | 40.9 |
| ID102595860 | | 35 | | 36.2 | | 36.7 | | 42.4 | | 32.4 | | 34.5 | | | 31.3 | | 29.2 | | 29.9 | | 45.1 | | 42.2 | | 47.4 | | 47.1 | | 44.2 | | 45.1 | | 45.4 | | | 47.4 | | 45 | 45.7 |
| ID102578898 | | 34.6 | | 34.61 37.9 | | | | 41.7 | | 32.4 | | 35.8 | | | 32 | | 31.3 | | 33.1 | | 44.9 | | 42.5 | | 44.3 | | 45.3 | | 43.5 | | 42.6 | | 43.1 | | | 45.1 | | 43.1 | 41.4 |
| ID102578810 | | 35.3 | | 35.8 | | 36.4 | | 44.7 | | 32.4 | | 33.6 | | | 30.8 | | 31.5 | | 28.4 | | 46.5 | | 42.1 | | 44.7 | | 46.3 | | 43.8 | | 46.9 | | 46.2 | | | 49.3 | | 45.2 | 47.5 |
| ID102587248 | | 35.5 | | 36.3 | | 38.3 | | 45.2 | | 32.1 | | 32.5 | | | 31.4 | | 31.6 | | 29 | | 45.6 | | 41.7 | | 46.4 | | 46.1 | | 43.9 | | 47.2 | | 45.1 | | | 48.7 | | 44.2 | 45.4 |
| ID107820469 | | 36.5 | | 37.4 | | 46 | | 40.6 | | 31.6 | | 34.8 | | | 34.5 | | 31.5 | | 32.3 | | 33.5 | | 33.8 | | 33.7 | | 25.5 | | 26.2 | | 25 | | 25 | | | 23.4 | | 22.9 | 24.5 |
| ID107803828 | | 34.8 | | 34.8 | | 38.3 | | 42.8 | | 32.7 | | 33.7 | | | 29.7 | | 31.8 | | 29.4 | | 47.6 | | 43.5 | | 49.8 | | 47.5 | | 45.1 | | 47.9 | | 46.6 | | | 50.4 | | 46.9 | 49 |
| ID107824944 | | 32.9 | | 33.3 | | 37.2 | | 43.8 | | 32.4 | | 31 | | | 30.4 | | 31.5 | | 29.3 | | 47.5 | | 46 | | 49.3 | | 47.4 | | 45.5 | | 48.1 | | 47.6 | | | 50.8 | | 47.8 | 49.1 |
| **Supplementary table 4** (*Continued*) | | | | | | | | | | | | | | | | | | | | | | | | | | | | | | | | | | | | | | | |
| ID107823411 | 100 | |  |  |  | |  | |  | |  | |  |  | |  | |  | |  | |  |  |  | |  | |  | |  | |  | |  |  | |  |  |  |
| ID107801151 | 89.8 | | 100 |  |  | |  | |  | |  | |  |  | |  | |  | |  | |  |  |  | |  | |  | |  | |  | |  |  | |  |  |  |
| ID102605560 | 73.8 | | 75.6 | 100 |  | |  | |  | |  | |  |  | |  | |  | |  | |  |  |  | |  | |  | |  | |  | |  |  | |  |  |  |
| ID543987 | 67.1 | | 68.4 | 87.6 | 100 | |  | |  | |  | |  |  | |  | |  | |  | |  |  |  | |  | |  | |  | |  | |  |  | |  |  |  |
| ID102605428 | 53 | | 54.3 | 54.9 | 53.5 | | 100 | |  | |  | |  |  | |  | |  | |  | |  |  |  | |  | |  | |  | |  | |  |  | |  |  |  |
| ID102596927 | 49.4 | | 50 | 51 | 49.5 | | 68.2 | | 100 | |  | |  |  | |  | |  | |  | |  |  |  | |  | |  | |  | |  | |  |  | |  |  |  |
| ID107814850 | 49.7 | | 51.3 | 52.5 | 50.1 | | 72.8 | | 71.1 | | 100 | |  |  | |  | |  | |  | |  |  |  | |  | |  | |  | |  | |  |  | |  |  |  |
| ID824894 | 46.9 | | 47.3 | 47.7 | 44.7 | | 46.5 | | 44.1 | | 44 | | 100 |  | |  | |  | |  | |  |  |  | |  | |  | |  | |  | |  |  | |  |  |  |
| ID824893 | 43.6 | | 43.3 | 45.3 | 43.9 | | 44.5 | | 43.6 | | 42.6 | | 58.2 | 100 | |  | |  | |  | |  |  |  | |  | |  | |  | |  | |  |  | |  |  |  |
| ID824891 | 43.4 | | 43.4 | 44.8 | 43.2 | | 44.9 | | 43.4 | | 42.1 | | 57.2 | 66.5 | | 100 | |  | |  | |  |  |  | |  | |  | |  | |  | |  |  | |  |  |  |
| ID102601393 | 34.7 | | 34.7 | 34.5 | 32.7 | | 36.5 | | 33.9 | | 31.5 | | 30.3 | 29.1 | | 30.3 | | 100 | |  | |  |  |  | |  | |  | |  | |  | |  |  | |  |  |  |
| ID102588651 | 31.4 | | 32.7 | 31 | 28.7 | | 26.9 | | 33.3 | | 28.3 | | 36.6 | 32.7 | | 34.8 | | 39.4 | | 100 | |  |  |  | |  | |  | |  | |  | |  |  | |  |  |  |
| ID107784423 | 46.6 | | 46.8 | 48.5 | 47.7 | | 48.3 | | 46.2 | | 47.3 | | 39.8 | 43.6 | | 42 | | 41.5 | | 32.7 | | 100 |  |  | |  | |  | |  | |  | |  |  | |  |  |  |
| ID102581946 | 39.4 | | 40.2 | 42.1 | 38.6 | | 43.5 | | 41.2 | | 40.2 | | 39.6 | 37.6 | | 38.8 | | 41.7 | | 42.4 | | 46.5 | 100 |  | |  | |  | |  | |  | |  |  | |  |  |  |
| ID102595860 | 41.2 | | 41.8 | 44.6 | 41.6 | | 43 | | 42.9 | | 45 | | 41.7 | 40.5 | | 40.8 | | 40.2 | | 48.2 | | 48.1 | 45.4 | 100 | |  | |  | |  | |  | |  |  | |  |  |  |
| ID102578898 | 42.7 | | 44.4 | 44.3 | 42 | | 44 | | 40.5 | | 41.2 | | 39.8 | 38.7 | | 38.7 | | 46.2 | | 45.8 | | 48 | 47.1 | 49.6 | | 100 | |  | |  | |  | |  |  | |  |  |  |
| ID102578810 | 42.8 | | 44.7 | 45.5 | 43 | | 43.3 | | 43.9 | | 45.5 | | 41.1 | 38.2 | | 38.5 | | 44.4 | | 44.2 | | 48.5 | 45.9 | 45.6 | | 47.9 | | 100 | |  | |  | |  |  | |  |  |  |
| ID102587248 | 43.5 | | 43.9 | 47.8 | 43.6 | | 44.1 | | 44.7 | | 45.8 | | 42.9 | 39.4 | | 41 | | 48.3 | | 47.4 | | 48.8 | 49.3 | 49.9 | | 52.6 | | 62.5 | | 100 | |  | |  |  | |  |  |  |
| ID107820469 | 28.9 | | 29.3 | 29.1 | 30.6 | | 24.4 | | 27 | | 36.1 | | 32.9 | 26.5 | | 28 | | 45.5 | | 46.2 | | 38.6 | 42.9 | 43.1 | | 44.1 | | 50.2 | | 62.7 | | 100 | |  |  | |  |  |  |
| ID107803828 | 45.1 | | 44.7 | 48.5 | 45.6 | | 45.2 | | 45 | | 46.8 | | 41.7 | 41.1 | | 41.1 | | 48.5 | | 46.5 | | 48.8 | 46 | 47.7 | | 48.3 | | 57.5 | | 73.6 | | 90.6 | | 100 |  | |  |  |  |
| ID107824944 | 47.6 | | 47.6 | 47.4 | 46.3 | | 45.4 | | 45.9 | | 46.2 | | 42.1 | 44.4 | | 44.3 | | 45 | | 39 | | 49.4 | 47.7 | 51.5 | | 50 | | 62 | | 78.1 | | 70 | | 95.2 | 100 | |  |  |  |
